# *BRCA1* promoter hypermethylation, 53BP1 protein expression and PARP-1 activity as biomarkers of DNA repair deficit in breast cancer

**DOI:** 10.1186/1471-2407-13-523

**Published:** 2013-11-05

**Authors:** William Jacot, Simon Thezenas, Romain Senal, Cathy Viglianti, Anne-Claire Laberenne, Evelyne Lopez-Crapez, Frédéric Bibeau, Jean-Pierre Bleuse, Gilles Romieu, Pierre-Jean Lamy

**Affiliations:** 1 Department of Medical Oncology, Montpellier Cancer Institute, Montpellier, France; 2 Translational Research Unit, Montpellier Cancer Institute, 208 rue des Apothicaires, 34298 Montpellier Cedex 5, France; 3 Department of Biostatistics, Montpellier Cancer Institute, Montpellier, France; 4 Department of Biology and Oncogenetic, Montpellier Cancer Institute, Montpellier, France; 5 Department of Pathology, Montpellier Cancer Institute, Montpellier, France

**Keywords:** Breast cancer, PARP-1, 53BP1, BRCA, Methylation

## Abstract

**Background:**

Poly(adenosine diphosphate–ribose) polymerase 1 (PARP-1) and the balance between BRCA1 and 53BP1 play a key role in the DNA repair and cell stress response. PARP inhibitors show promising clinical activity in metastatic triple negative (TN) or *BRCA*-mutated breast cancer. However, a comprehensive analysis of PARP-1 activity, *BRCA1* promoter methylation and 53BP1 expression in tumours without known *BRCA1* mutation has not yet been carried out.

**Methods:**

We investigated cytosolic PARP-1 activity, 53BP1 protein levels and *BRCA1* promoter methylation in 155 surgical breast tumour samples from patients without familial breast cancer history or known *BRCA1* mutations who were treated between January 2006 and November 2009 and evaluated their statistical association with classical predictive and prognostic factors.

**Results:**

The mitotic count score was the only parameter clearly associated with PARP-1 activity. *BRCA1* promoter hypermethylation (15.4% of all cancers) was significantly associated with uPA and PAI-1 levels, tumour grade, mitotic count score, hormone receptor and HER2 negative status and TN profile (29% of TN tumours showed *BRCA1* promoter hypermethylation compared to 5% of grade II-III hormone receptor-positive/HER2-negative and 2% of HER2-positive tumours). No statistical association was found between *BRCA1* promoter hypermethylation and PARP-1 activity. High 53BP1 protein levels correlated with lymph node positivity, hormone receptor positivity, molecular grouping, unmethylated *BRCA1* promoter and PARP-1 activity. In TN tumours, *BRCA1* promoter methylation was only marginally associated with age, PARP-1 activity was not associated with any of the tested clinico-pathological factors and high 53BP1 protein levels were significantly associated with lymph node positivity. Only 3 of the 14 TN tumours with *BRCA1* promoter hypermethylation presented high 53BP1 protein levels.

**Conclusions:**

Breast cancers that harbour simultaneously high 53BP1 protein level and *BRCA1* promoter hypermethylation and are the putative target population of drugs targeting DNA repair appear to be restricted to a small subgroup of TN tumours.

## Background

Up-regulation of their DNA repair capacity represents a common mechanism used by cancer cells to survive DNA-damaging therapy [1]. Lack of efficient DNA repair by simultaneous loss or inhibition of two DNA repair pathways causes synthetic lethality and cell death, thus representing an attractive approach for cancer therapy [2]. For instance, *BRCA*-deficient cancer cells, in which DNA double strand break repair (DSB) by homologous recombination is deficient [3,4], are particular sensitive to treatment with inhibitors of Poly(ADP-ribose) (PAR) polymerase 1 (PARP-1), a nuclear enzyme that recognizes and facilitates repair of DNA damage induced by oxidation, alkylation and ionizing radiation [2,5-7], showing reduced clonogenic survival and DNA DSB repair defects [8,9]. Moreover, the persistent single-strand breaks (SSB) formed upon PARP-1 inhibition cannot be repaired effectively in the absence of functional BRCA1 or BRCA2, resulting in accumulation of chromosomal abnormalities, cell cycle arrest and apoptosis [8,9]. Thus, PARP-1 may be an important target for *BRCA-*deficient breast cancer chemotherapy [8-11], as emphasized also by the clinical activity of the PARP inhibitor (PARP*i*) olaparib in patients with *BRCA*-mutated breast cancer [3]. Up-regulation of PARP-1 expression and activity has been observed in a variety of human tumours [12,13]. In breast cancer, PARP-1 up-regulation has been associated with decreased survival [14] and triple-negative (TN) cancers (breast tumours in which estrogen receptor [ER], progesterone receptor [PR] and human epidermal growth factor receptor 2 [HER2] are not expressed) [15]. None of these studies considered PARP-1 activity together with BRCA1 functional status, except in the case of *BRCA1-*mutated cancers, which represent only around 5% of all breast cancers [16-18]. Loss of BRCA1 nuclear expression correlates with high tumour grade (*p* < 0.025) and ER-negative tumours. Absence or reduced BRCA1 expression in tumours without *BRCA1* mutations appears linked to hypermethylation of the *BRCA1* promoter region [19], a condition reported in 9.1–37% of sporadic breast cancers and associated with infiltrating ductal type, high (grade II-III) tumour grade, ER negativity, basal markers expression, younger age at diagnosis, low *BRCA1* mRNA expression and marked reduction or loss of BRCA1 protein expression [19-25]. Thus, *BRCA1* promoter hypermethylation could be a marker of BRCA1 deficiency in the absence of *BRCA1* mutation, as these two events appears mutually exclusive [24].

Some conditions, such as a loss of P53 binding protein 1 (53BP1, a protein involved in DNA damage checkpoint activation and DNA repair), could allow cells to tolerate BRCA1 deficiency. 53BP1 localizes to sites of DNA DSBs, promotes non-homologous end joining (NHEJ)-mediated repair and checkpoint activation and inhibits homologous recombination [26-29]. As BRCA1 promotes homologous recombination, it might counteract 53BP1 effect [30,31]. Thus, the balance between 53BP1 and BRCA1 regulates the competition between the NHEJ and homologous recombination pathways in DNA DSB repair [32]. In *BRCA1* mutant/inactivated cells, repair by homologous recombination is defective and the error-prone NHEJ predominates, resulting in high sensitivity to DNA-damaging agents and PARP*i*. However, when both BRCA1 and 53BP1 are lost, repair by homologous recombination is restored and the sensitivity to DNA damaging agents is reduced, leading to resistance to cis-platinum and PARP*i* in *BRCA1*-deficient cells, suggesting a critical role of 53BP1 in cancer cells in which *BRCA1* is mutated or epigenetically silenced [30-33]. Reduced 53BP1 expression has been reported in sporadic basal-like, TN and *BRCA*-mutated breast cancers [30]. It thus appears important to simultaneously evaluate 53BP1 status and *BRCA1* mutation/promoter methylation to precisely estimate homologous recombination functionality in breast tumours.

Many PARP*i* are presently in pre-clinical or clinical development, preferentially for patients with *BRCA*-deficient tumours or TN breast cancers, due to the over-representation of this breast cancer subtype in patients with *BRCA* mutations. However, there is no validated screening test to identify the patients who may receive the most benefit from PARP*i*. Recent data show that most of the non-*BRCA*-mutated TN breast cancers do not benefit from such drugs, while some non-TN *BRCA*-mutated tumours could respond to PARP*i*[34]. Moreover, two different groups [35,36] recently reported that breast cancers with epigenetically silenced *BRCA1* are sensitive to PARP*i* monotherapy, providing robust evidence to support the use of PARP*i* in the treatment of selected sporadic *BRCA1*-inactivated breast cancers. A comprehensive analysis of the PARP-1/BRCA1/53BP1 factors of DNA repair in the different breast cancer subtypes could enable this selection and promote the use of these compounds outside the TN subtype.

Here, we comprehensively and simultaneously evaluated the BRCA1/53BP1/PARP-1 repair network in three groups (HER2-positive, grade II-III hormone receptor [HR]-positive/HER2-negative and TN) of sporadic breast cancers (n = 155) from patients without familial breast cancer history or known *BRCA1* mutations to identify tumour population(s) with a theoretically high susceptibility to PARP*i*.

## Methods

### Patients and tumour samples

This is a retrospective monocentric study using samples from a research-dedicated tumour biobank (cytosol and DNA samples). A total of 556 consecutive patients with breast cancer referred to the Montpellier Cancer Institute between January 2006 and November 2009 were prospectively entered in the biobank database. The DNA collection was created using frozen, histologically proven and macro-dissected invasive breast cancer specimens that were primarily handled for uPA/PAI-1 testing [37]. Tumour samples dedicated to the molecular analysis were selected based on the immediate diagnosis by using frozen sections. Additional tumour tissue samples were then chosen after the definitive histological diagnosis (with quantification of the percentage of tumour cells) and grade assessment after fixation. This could be possible because frozen and formalin-fixed tumour tissue samples were selected from the same tumour areas. Only samples that contained at least 50% of tumour cells were used for uPA/PAI-1 testing. ER and PR protein expression was assessed by IHC using the anti-ER (clone 6 F11, 1:100, Leica Biosystems, United Kingdom) or anti-PR (clone PgR636, 1:400, Dako, Denmark) mouse monoclonal antibodies respectively. Tumours were considered as ER- and PR-positive when more than 10% of tumour cells were stained by immunohistochemistry (IHC). HER2 protein expression was assessed by IHC using the A485 monoclonal antibody (Dako, Denmark). Breast cancers with HER2 scores of 0 and 1+ were considered negative. Gene amplification was evaluated in HER2 2+ tumours using FISH or CISH. HER2 3+ tumours were considered as positive. Grade scoring, using the Scarf, Bloom and Richardson scoring method, modified as proposed by Elston and Ellis [38], was performed to score all tumours. For this study, 155 sporadic breast tumours from patients without familial breast cancer history or known *BRCA1* mutations were selected. Tumours were classified in three groups (grade II-III HR-positive/HER2-negative, n = 57; HER2-positive, n = 50; or TN, n = 48) that were matched for age, T and N status. This study was reviewed and approved by the Montpellier Cancer Institute Review Board. All patients gave their written, informed consent. Although this was not a prognostic study, it followed the REMARK guidelines to enable future evaluation of the prognostic impact of the evaluated factors [39].

### Tissue processing and DNA extraction

Each frozen tumour tissue sample was pulverized in liquid nitrogen with a grinder (Cryobroyeur-2000P Automatique, Rivoire, Montpellier, France) and then homogenized with a Polytron homogenizer (Glen Mills, Clifton, NJ) using a Triton buffer/tissue ratio of 10:1 (vol/wt; Triton buffer 1%, 2 mL 10% Triton X-100 in 18 mL of Tris -buffered Saline [TBS, 50 mM Tris, 150 mM NaCl], pH 8.5) [37]. Homogenates were centrifuged at 10000 × g for 15 minutes. The supernatants were used to prepare cytosols and the total protein content was quantified using the Pierce assay (BCA Protein Assay Kit, Pierce Biotechnology, Rockford, IL) as previously described [37]. Total genomic DNA was extracted from the pellets using the QIAamp DNA Mini Kit (Qiagen GmbH, Hilden, Germany) according to the manufacturer’s protocol. DNA yield and purity were assessed using the Nanodrop (Thermo Fisher Scientific, Waltham, USA) by measuring the absorbance at 260 nm and 280 nm. All samples had a 260/280 nm ratio higher than 1.7. DNA was stored at −20°C in TE buffer (10 mM Tris and 0.5 mM EDTA, pH 7.6).

### PARP-1 activity

The Trevigen HT Universal 96-well PARP Assay Kit (*HT Universal Colorimetric PARP Assay Kit with Histone-coated Strip Wells, Trevigen, Gaithersburg, MD, USA*) assesses cytosolic PARP-1 activity by measuring the incorporation of biotinylated poly(ADP-ribose) onto histone proteins in a 96-well strip format. 50 μl of 1× PARP Buffer was added to rehydrate the histone-coated wells for 30 minutes and then removed. The PARP-HSA standard was used to obtain a standard curve with an activity range from 1 mU to 1 U. Cytosol samples were diluted in PARP Buffer in order to contain at least 20 μg of protein and 25 μL were added in each well. Then, 25 μl of 1× PARP Cocktail (obtained by diluting 25 μL of 10× PARP Cocktail and 25 μL of 10× Activated DNA in 1× PARP buffer) were added to each well and incubated at room temperature for 60 minutes. After two washes with 200 μL 1× PBS + 0.1% Triton X-100 and two washes with 200 μL 1× PBS, 50 μL of 1× Strep-HRP was added and incubated at room temperature for 60 minutes. Wells were washed as before and 50 μL of pre-warmed TACS-Sapphire substrate was added and incubated in the dark at room temperature for 15 minutes. Reactions were stopped with 50 μL 0.2 M HCl. Absorbance was read at 450 nm and the concentration values of the diluted samples were calculated from the standard curves and expressed in U/mL. PARP-1 activity was normalized to the protein concentration and results were expressed in U/mg of protein (U/mgP).

### BRAC1 promoter methylation status

DNA methylation patterns at the CpG islands of the *BRCA1* promoter were assessed using a methylation-specific PCR assay [40]. This method distinguishes unmethylated and methylated alleles on the basis of sequence changes following bisulphite treatment of DNA that converts only unmethylated cytosines to uracil. Bisulphite treatment was performed using the EpiTect Bisulfite Kit (QIAGEN GmbH, Hilden, Germany). PCRs were performed on an Eppendorf Mastercycler® apparatus (Eppendorf, Hamburg, Germany) with the EpiTect MSP-PCR Kit (QIAGEN GmbH, Hilden, Germany) and specific primers designed for methylated or unmethylated *BRCA1* DNA sequences [40]. EpiTect PCR Control DNA Set (Qiagen Hindel, Germany) containing both bisulfite converted methylated and unmethylated DNA and unconverted unmethylated DNA were also added as MS-PCR controls. Seven μL of each PCR product was loaded directly onto 1% agarose + 3% Nusieve GTG agarose gel, stained with 1 μL/10 ml SYBR® Safe DNA gel stain and visualized under UV light.

### 53BP1 protein quantification

53BP1 concentration in the tumour cytosol samples was determined using the TP53BP1 ELISA kit (Cusabio, Wuhan, Hubei Province 430223, P.R.China). Protein concentration in cytosols ranged from 0.5 to 20 mg/mL. For 53BP1 quantification 100 μL of pure cytosol were used for each sample. 100 μl of each sample and standards were incubated at 37°C for 2 hours to allow binding of 53BP1 to the immobilized anti-TP53BP1 antibody. After removal of unbound material without washing, each well was incubated at 37°C with 100 μL of a biotin-conjugated antibody specific for TP53BP1 for one hour. After three washes, avidin-conjugated Horseradish Peroxidase (HRP) was added at 37°C for one hour. Following a wash to remove any unbound avidin-HRP, 90 μl of TMB substrate solution was added for 30 min. 50 μl of Stop Solution was added into each well and absorbance was read at 450 nm with an MRX spectrophotometer (Dynatech laboratories). The range of standardization goes from 6.25 pg/ml to 400 pg/ml with a limit of detection of 2 pg/ml. 53BP1 levels were standardized to the total protein content and results expressed in pg/mgP.

### Statistical methods

In this monocentric retrospective study, our main goal was to evaluate the correlations of clinico-pathological features with PARP-1 activity, 53BP1 expression and *BRCA1* promoter hypermethylation. Categorical variables (all parameters precluding their concomitant use in adjuvant decision making) were reported by means of contingency tables. To investigate the association of classical clinico-pathological parameters with PARP-1 activity, 53BP1 protein level and *BRCA1* gene promoter methylation, univariate analyses were performed for categorical variables using the Pearson’s chi-square test or the Fisher’s exact test when applicable. For continuous variables, medians and ranges were computed. The non-parametric Kruskal-Wallis test or the Mann Whitney test were used, as appropriate, to evaluate significant differences between groups of interest. Spearman’s correlation was performed to investigate the strength of the relationship between pairs of variables. The Kaplan-Meier method was used to estimate the survival rates from the date of surgery until the date of the event of interest. Median survivals were presented with 95% confidence interval (95% CI). For OS, the event was death whatever the cause. Patients lost to follow-up were censored at the date of the last documented visit. For RFS, the event was recurrence. Patients alive at the last follow-up without recurrence were censored at the last follow-up date. Patients who died without recurrence were censored at the date of death. All *p* values reported are two-sided and the significance level was set at 5% (p < 0.05). Statistical analysis was performed using the STATA 11 software (Stata Corporation, College Station, TX).

## Results

### Patients’ and tumours’ characteristics

A total of 155 patients with breast cancers that were classified in three molecular (HER2-positive, HR-positive / HER2-negative and TN) groups were selected for this study. The median age was 54 years (range 29–75 years). The main clinico-pathological characteristics of the population are summarized in Table 1. As only one tumour was classified as grade I and tubule formation score 1 and none as nuclear pleomorphism score 1, tumours with grade I and II and tubule formation scores 1 and 2 were grouped for statistical analyses.

**Table 1:** Patients and tumours characteristics

		**PARP activity (Low < 7 U / mg Protein, High ≥ 7 U / mg Protein)**					***BRCA1 *** **methylation status**		
**Patients’ characteristics**	**N (%)**	**Low n (%)**	**High n (%)**	***p***	**Mean ± SD**	***p***	**Methylated n (%)**	**Not Methylated n (%)**	***p***
**Age at diagnosis (years)**				0.92		0.83			0.17
Median (range)	54 (29–75)								
≤ 54	80 (51.6%)	38 (52.1%)	42 (51.2%)		11.7 (13)		12 (66.7%)	68 (49.6%)	
> 54	75 (48.4%)	35 (47.9%)	40 (48.8%)		12.7 (20.5)		6 (33.3%)	69 (50.4%)	
**Menopausal status**				0.87		0.54			0.62
Pre-menopausal	69 (44.5%)	33 (45.2%)	36 (43.9%)		12.5 (13.8)		9 (50.0%)	60 (43.8%)	
Post-menopausal	86 (55.5%)	40 (54.8%)	46 (56.1%)		11.9 (19.3)		9 (50.0%)	77 (56.2%)	
**T classification**				0.67		0.60			0.76
T1	76 (49%)	36 (49.3%)	40 (48.8%)		14 (21.1)		9 (50.0%)	67 (48.9%)	
T2	75 (48.4%)	36 (49.3%)	39 (47.6%)		10.4 (12)		9 (50.0%)	66 (48.2%)	
T3-4	4 (2.6%)	1 (1.4%)	3 (3.7%)		10 (3.7)		0	4 (2.9%)	
**N classification**				0.75		0.8			0.71
N0	106 (68.4%)	49 (67.1%)	57 (69.5%)		12.4 (18.1)		13 (72.2%)	93 (67.9%)	
N+	49 (31.6%)	24 (32.9%)	25 (30.5%)		11.8 (14.6)		5 (27.8%)	44 (32.1%)	
**Histology**				0.27		0.3			0.53
Ductal	120 (77.4%)	53 (72.6%)	67 (81.7%)		13.1 (18.3)		15 (83.3%)	105 (76.6%)	
Lobular	9 (5.8%)	4 (5.5%)	5 (6.1%)		7.8 (8.3)		0	9 (6.6%)	
Other	26 (16.8%)	16 (21.9%)	10 (12.2%)		9.6 (12.5)		3 (16.7%)	23 (16.8%)	
**Grade**				0.23		0.02			0.03
I / II	1 (0.6%) / 51 (32.9%)	28 (38.4%)	24 (29.3%)		9.2 (12.8)		2 (11.1%)	50 (36.5%)	
III	103 (66.5%)	45 (61.6%)	58 (70.7%)		13.7 (18.7)		16 (88.9%)	87 (63.5%)	
**Mitotic count score**				0.04		0.003			0.04
1	31 (20%)	20 (27.4%)	11 (13.4%)		8.3 (13.6)		1 (5.6%)	30 (21.9%)	
2	62 (40%)	30 (41.1%)	32 (39.0%)		10.8 (16.2)		5 (27.8%)	57 (41.6%)	
3	62 (40%)	23 (31.5%)	39 (47.6%)		15.6 (18.8)		12 (66.7%)	50 (36.5%)	
**ER**				0.88		0.66			0.001
Positive	88 (56.8%)	41 (56.2%)	47 (57.3%)		12.1 (16.9)		4 (22.2%)	84 (61.3%)	
Negative	67 (43.2%)	32 (43.8%)	35 (42.7%)		12.4 (17.3)		14 (77.8%)	53 (38.7%)	
**PR**				0.94		0.75			0.01
Positive	59 (38.1%)	28 (38.4%)	31 (37.8%)		11.5 (14.3)		2 (11.1%)	57 (41.6%)	
Negative	96 (61.9%)	45 (61.6%)	51 (62.2%)		12.6 (18.6)		16 (88.9%)	80 (58.4%)	
**HER2**				0.62		0.5			0.01
Positive	50 (32.3%)	25 (34.2%)	25 (30.5%)		9.7 (11.3)		1 (5.6%)	49 (35.8%)	
Negative	105 (67.7%)	48 (65.8%)	57 (69.5%)		13.4 (19.1)		17 (94.4%)	88 (64.2%)	
**Molecular profile grouping**				0.81		0.68			<0.001
HER2+	50 (32.3%)	25 (34.2%)	25 (30.5%)		9.7 (11.3)		1 (5.6%)	49 (35.8%)	
HR+/HER2-	57 (36.7%)	25 (34.2%)	32 (39.0%)		13.4 (19.9)		3 (16.7%)	54 (39.4%)	
Triple negative	48 (31%)	23 (31.5%)	25 (30.5%)		13.4 (18.3)		14 (77.8%)	34 (24.8%)	
**uPA level ( ≥3)**				0.37		0.3			0.01
High	97 (62.6%)	43 (58.9%)	54 (65.9%)		12.9 (17)		16 (88.9%)	81 (59.1%)	
Low	58 (37.4%)	30 (41.1%)	28 (34.1%)		11 (17.2)		2 (11.1%)	56 (40.9%)	
**PAI-1 level ( ≥14)**				0.32		0.42			0.03
High	112 (72.3%)	50 (68.5%)	62 (75.6%)		11.4 (12.4)		17 (94.4%)	95 (69.3%)	
Low	43 (27.7%)	23 (31.5%)	20 (24.4%)		14.2 (25.5)		1 (5.6%)	42 (30.7%)	
**PARP Activity**									0.82
≤ 2.6 U/mg Prot	39 (25.2%)	-	-	-	-	-	4 (22.2%)	35 (25.5%)	
2.7 - 7	42 (27.1%)	-	-	-	-	-	6 (33.3%)	36 (26.3%)	
7.1 - 14	37 (23.9%)	-	-	-	-	-	3 (16.7%)	34 (24.8%)	
> 14	37 (23.9%)	-	-	-	-	-	5 (27.8%)	32 (23.4%)	

### PARP-1 activity

**Figure 1:**
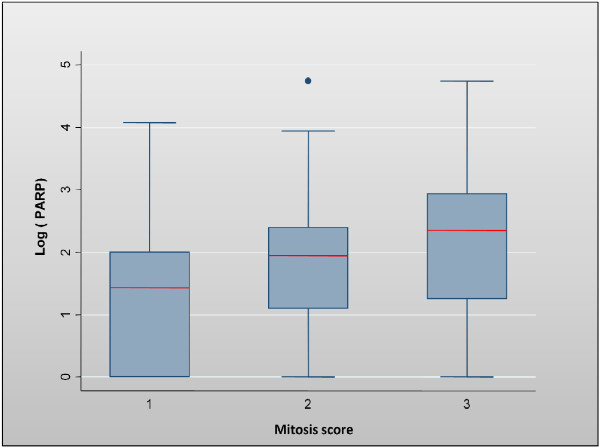
Correlation between PARP cytosolic level (logarithmic scale) and the mitotic count score.

The mean PARP-1 activity (U/mg of cytosolic protein) was 12.2 (standard deviation: 17.02), with a median of 7.0 (range: 1.0 to 114.2). No significant difference was observed in the three tumour groups concerning PARP-1 activity. Only the mitotic count score was clearly correlated with PARP-1 activity, using either the mean (*p* = 0.007), median (Figure 1 and Table 1) or the upper quartile limit (*p* = 0.03) as cut-off values. In addition, grade significantly (*p* = 0.02) correlated with PARP-1 activity using the mean as cut-off value. Using the mitotic count score as a continuous variable, a weak correlation was found between the number of mitoses and PARP-1 cytosolic activity (Spearman correlation coefficient: 0.234, *p* = 0.003).

### BRCA1 promoter methylation

Bisulphite treatment was successfully performed for all samples. *BRCA1* promoter hypermethylation was detected in 18 tumours (Additional file 1: Table S1) and was significantly associated with the TN status. Indeed, in 29% (14/48) of TN breast tumours *BRCA1* promoter was hypermethylated compared to 5% (3/57) of HR-positive/HER2-negative and 2% (1/50) of HER2-positive tumours (Table 1). *BRCA1* promoter hypermethylation was significantly associated also with uPA and PAI-1 levels, grade and mitotic count score and ER-, PR- or HER2-negative status. No statistical association was found between *BRCA1* promoter hypermethylation and PARP-1 cytosolic activity.

### 53BP1 protein expression level

53BP1 protein expression could not be determined in three tumours, due to insufficient amount of biological sample. The mean 53BP1 protein level in the remaining 152 tumours was 12.5 pg/mgP (median: 9.6 pg/mgP; range: 2.0-93.0 pg/mgP) (Table 2). High (≥9.6 pg/mgP) 53BP1 levels correlated with molecular grouping (63.2% of HR-positive/HER2-negative *vs*. 47.9% of HER2-positive and 36.2% of TN tumours, *p* = 0.022), lymph node positivity (43.3% of N0 *vs*. 64.6% of N1+ tumours, *p* = 0.015), ER positivity (59.8% of ER-positive *vs*. 36.9% of ER-negative tumours, *p* = 0.005), PR positivity (62.7% of PR-positive *vs*. 41.9% of PR-negative cancers, *p* = 0.013), unmethylated *BRCA1* promoter (53% of unmethylated *vs*. 27.8% of methylated cancers, *p* = 0.045) and PARP-1 activity (60.8% of tumours with high (≥7 U/mg Prot) PARP-1 activity *vs*. 38.4% of tumours with low (<7 U/mg Prot) PARP-1 activity, *p* = 0.006 using PARP-1 median value as a cut-off; *p* = 0.048 categorizing PARP-1 values as quartiles [Table 2]). No correlation was found between PARP-1 activity and 53BP1 levels using continuous variables (Additional file 2: Figure S1). Both high 53BP1 levels and *BRCA1* promoter hypermethylation were observed in three TN tumours and two non-TN tumours (Additional file 1: Table S1).

**Table 2:** 53BP1 protein expression level and correlation with clinico-pathological parameters

		**53BP1 protein expression level (Low < 9.6 U / mg Protein, High ≥9.6 pg/mg Protein)**		
**Patients’ characteristics**	**N (%)**	**Low n (%)**	**High n (%)**	***p***
**Age at diagnosis (years)**				1
Median (range)	54 (29–75)			
≤ 54	80 (52.6%)	40 (52.6%)	40 (52.6%)	
> 54	72 (47.4%)	36 (47.4%)	36 (47.5%)	
**Menopausal status**				0.87
Pre-menopausal	69 (44.7%)	34 (44.7%)	35 (46.1%)	
Post-menopausal	83 (55.3%)	42 (55.3%)	41 (53.9%)	
**T classification**				0.57
T1	75 (49.3%)	37 (48.7%)	38 (50%)	
T2	73 (48%)	38 (50%)	35 (46.1%)	
T3-4	4 (2.6%)	1 (1.3%)	3 (3.9%)	
**N classification**				0.015
N0	104 (68.4%)	59 (77.6%)	45 (59.2%)	
N+	48 (31.6%)	17 (22.4%)	31 (40.8%)	
**Histology**				0.44
Ductal	117 (77%)	58 (76.4%)	59 (77.6%)	
Lobular	9 (5.9%)	3 (3.9%)	6 (7.9%)	
Other	26 (17.1%)	15 (19.7%)	11 (14.5%)	
**Grade**				0.46
I / II	1 (0.7%) / 50 (32.9%)	23 (30.3%)	28 (36.8%)	
III	101 (66.4%)	53 (69.7%)	48 (63.2%)	
**ER**				0.005
Positive	87 (57.2%)	35 (46.1%)	52 (68.4%)	
Negative	65 (42.8%)	41 (53.9%)	24 (31.6%)	
**PR**				0.013
Positive	59 (38.8%)	22 (28.9%)	37 (48.7%)	
Negative	93 (61.2%)	54 (71.1%)	39 (51.3%)	
**HER2**				0.73
Positive	48 (31.6%)	25 (32.9%)	23 (30.3%)	
Negative	104 (68.4%)	51 (67.1%)	53 (69.7%)	
**Molecular profile grouping**				0.022
HER2+	48 (31.6%)	25 (32.9%)	23 (30.3%)	
HR+/HER2-	57 (37.5%)	21 (27.6%)	36 (47.4%)	
Triple Negative	47 (30.9%)	30 (39.5%)	17 (22.4%)	
***BRCA1 *** **methylation Status**				0.045
Methylated	18 (11.8%)	13 (17.1%)	5 (6.6%)	
Not Methylated	134 (88.2%)	63 (82.9%)	71 (93.4%)	
**PARP activity**				0.048
≤ 2.6 U/mg Prot	39 (25.6%)	21 (27.6%)	18 (23.8%)	
2.7 - 7	41 (27%)	27 (35.5%)	14 (18.4%)	
7.1 - 14	36 (23.7%)	14 (18.4%)	22 (28.9%)	
> 14	36 (23.7%)	14 (18.4%)	22 (28.9%)	

### The BRCA1 / 53BP1/ PARP-1 pathway in triple negative breast cancers

*BRCA1* promoter methylation status, 53BP1 protein levels and PARP-1 activity in the 48 TN breast cancers and their clinico-pathologically data are presented in Additional file 3: Table S2. In this group, only age was almost negatively associated with *BRCA1* promoter methylation (83.3% of cancers were unmethylated in patients >54 *vs*. 58.3% in patients ≤54 years, *p* = 0.057). PARP-1 activity was not associated with any of the tested clinico-pathological features. High 53BP1 levels were significantly associated with lymph node positivity (24.2% of N0 *vs*. 64.3% of N1+ cancer, *p* = 0.009). The association of high 53BP1 and PAI-1 protein levels was almost significant (43.2% of cancers with high *vs*. 10% of cancer with low PAI-1 protein levels, *p* = 0.052). Only three of the 14 tumours with *BRCA1* promoter hypermethylation had high 53BP1 protein levels. No clinico-pathological criterion could specifically identify this breast cancer population.

As recommendations regarding ER and PR cut-offs are not clearly established worldwide, we used an alternative, North American, 1% cut-off to define ER and PR positivity / negativity. Using this 1% threshold, the results were not significantly modified even if 2 TN cases were reclassified as HR+/HER2- cases using this alternative cut-off (2 cases with a 1-9% ER status, none for PR status).

### Survival analyses

Survival data were updated on June 10, 2012. At this time, after a median follow-up of 43.6 months (range 1.9 – 75.7 months), only 2 cancer-related deaths and 2 relapses were recorded (2 TN patients). The median 3-year OS and RFS were 0.986 (95% CI 0.954 - 0.999) and 0.986 (95% CI 0.954 - 0.999), respectively. This low number of relapses and deaths could be explained by a relatively brief follow-up, altogether with the fact that most of the tumours were small (pT1) and/or node negative tumours. In addition, considering the TN population, nearly all of the patients of this study received adjuvant chemotherapy. Even if the 2 events occurred in the TN population, the low number of events precludes a statistically robust analysis.

## Discussion

This study reports a comprehensive analysis of the BRCA/53BP1/PARP-1 factors of DNA repair in the largest cohort of patients with sporadic breast cancer to date. Clinical studies are currently under way to evaluate the efficacy of PARP*i* in patients with TN breast cancer. However, triple negativity alone does not appear to be a good surrogate marker for PARP*i* clinical sensitivity [34] as important biological differences exist within this group of tumours. Moreover, it is important to know whether sub-population of HR-positive and HER2-positive patients might also be eligible for such therapy.

We found that PARP-1 activity correlated only with the mitotic count score, without statistical association with *BRCA1* promoter hypermethylation. Using IHC, von Minckwitz *et al*. retrospectively evaluated the predictive and prognostic value of cytoplasmic (cPARP) and nuclear PARP (nPARP) expression in 638 pre-treatment biopsies from neoadjuvant anthracycline/taxane-treated patients [13]. High cPARP expression was significantly correlated with non-lobular histology, undifferentiated grade, positive nodal and negative HR status, but not with the HER2 status. Expression of cPARP was high in 35.5% of TN tumours, 24.6% of HER2-positive tumours and 18.0% of HR-positive/HER2-negative tumours. High cPARP expression was predictive of the achievement of pathologic complete response, particularly in HR-positive and HER2-negative tumours, and was a negative, but not independent prognostic factor of disease-free and overall survival. No correlation was found for nPARP expression. Ozretic *et al*. [41] investigated PARP expression in breast cancers with *BRCA1* (n = 66) or *BRCA2* (n = 27) mutations and in 53 sporadic breast cancers. Although they used the same PARP antibody described by von Minckwitz *et al*. [13], they did not observe significant cPARP staining. Conversely, nPARP expression was significantly increased in cancers with *BRCA1* or *BRCA2* mutations compared to sporadic tumours. No significant increase in nPARP expression was observed in the few sporadic TN breast cancers of their cohort. Their results suggest that nPARP and not cPARP expression is associated with BRCA-dependent DNA repair deficiency. However, their results cannot be extrapolated to the whole population of sporadic TN breast tumours due to the limited sample size. The results of the study by Rojo *et al*. [14] are consistent with the findings by Ozteric *et al*. They quantitatively evaluated nPARP-1 expression using a specific IHC signal intensity scanning assay in a range of normal to malignant breast lesions, including 330 patients treated for early breast cancer. nPARP-1 was overexpressed in about a third of ductal carcinoma in situ and infiltrating breast cancers and was associated with higher tumour grade, ER-negative tumours and TN phenotype. In this study, Ki-67 staining was used instead of mitotic count. As discrepancies are common between these two methods of proliferation evaluation, [42] a parallel cannot be drawn between this study and our results on this variable. Finally, multivariate analysis (median follow-up time: 4.8 years) indicated that nPARP-1 overexpression was an independent prognostic factor for both disease-free (HR 10.05; 95% CI 5.42–10.66) and overall survival (HR 1.82; 95% CI 1.32-2.52) [14]. These discordant results regarding the association of PARP quantification by IHC with prognosis could be linked to the fact that the IHC assay used for PARP determination detects both active and catalytically inactive, auto-modified PARP and not only functionally active PARP like in our study. However, to date, the question of the better way to evaluate tumoral PARP-1 activity (functional cytosolic assay as in our study, or morphological test such as IHC) is still open [13,14,41].

In our series, *BRCA1* promoter hypermethylation was found in 18 tumours and was significantly associated with a more aggressive clinico-biological profile and with triple negativity. Indeed, in 29% of TN tumours *BRCA1* promoter was hypermethylated compared to 5% of HR-positive/HER2-negative and 2% of HER2-positive tumours, consistent with the 36.7% reported by Veek *et al*. in 68 non-inherited TN breast cancers [36]. Altogether, these results suggest that the analysis of *BRCA1* hypermethylation could be included in the current and prospective PARP*i* clinical trials as a potential predictive biomarker. Wei *et al*. found a strong correlation between *ER* promoter and *BRCA1* promoter methylation, suggesting a higher frequency of *BRCA1* methylation in HR-negative breast cancers (no information was available on the HER2 status of these tumours) [43]. In the study evaluating the clinical impact of *BRCA1* promoter methylation in 135 Bulgarian HR-positive and HR-negative patients, Krasteva *et al*. reported that hypermethylation was present in 17.04% of the cases. Surprisingly, patients with *BRCA1* promoter hypermethylation displayed favourable clinical status as their tumours were smaller in size, lacked *p53* gene mutations and were of lobular type [44]. *BRCA1* promoter methylation was not significantly associated with ER, PR and HER2 status; however an evaluation of its association with the TN status was not reported. The presence of *BRCA1* promoter hypermethylation was not significantly associated with better overall survival (HR = 0.47, *p* = 0.2). No clear explanation of these discrepancies compared to other publications was proposed by the authors. No conclusion could be issued in our present study regarding the impact of these biomarkers status on survival, considering the relatively brief median follow-up of our population. However, this information will be studied later, after a significantly longer follow-up, allowing the occurrence of more events. Finally, we show that 53BP1 protein expression levels was significantly correlated with molecular grouping (63.2% of HR-positive/HER2-negative *vs*. 47.9% of HER2-positive and 36.2% of TN tumours) and unmethylated *BRCA1* promoter (53% of unmethylated *vs*. 27.8% of methylated cancers). Regarding definition of ER and PR positivity, recommendations regarding ER and PR cut-offs are not clearly established worldwide. We used in this study an European 10% cut-off to consider positive or negative ER and PR status [45]. This 10% cut-off can be considered as a standard of care in many countries. The 1% cut-off can be considered as another, North American, standard. However, our results were not significantly modified by the use of this 1% threshold for ER (2 TN cases) and PR positivity (no TN cases), and thus cannot be explained by the use of one or another ER/PR positivity threshold.

## Conclusions

In our study, the association of *BRCA1* promoter methylation and high 53BP1 protein levels was a rare event, even in the TN group. As this association appears to be the best situation to predict PARP*i* clinical activity (because loss of 53BP1 leads to partial restoration of homologous recombination and resistance to PARP*i*) [33] these results pledge for a strict selection of the target population of future trials involving these agents, and could, at the same time, explain the negative results of previous trials that did not include such strict selection [46]. A retrospective analysis of *BRCA1* promoter methylation and 53BP1 protein levels in the patients enrolled in such trials could help confirm the predictive impact of this tumour profile. In addition, evaluation of the 53BP1 protein levels in cases harbouring deleterious mutations in other less common homologous recombination genes with moderate penetrance, such as *PALB2*[47,48], need to be performed, as well as determination of the *PALB2* methylation status of this gene in *PALB2* non-mutated cases, as PALB2-deficient cells appears to be sensitive to PARPi [49].

## Abbreviations

53BP1: P53 binding protein 1; BRCA1: Breast cancer type 1 susceptibility protein; CISH: Chromogenic in situ hybridization; DNA: DeoxyriboNucleic acid; DSB: Double strand break; EDTA: EthyleneDiamineTetraacetic acid; ER: Estrogen receptor; FISH: Fluorescent in situ hybridization; Grade: Scarf, bloom and Richardson scoring method, modified as proposed by Elston and Ellis; HER2: Human epidermal growth factor receptor 2; HR: Hormone receptors; IHC: ImmunoHistoChemistry; NHEJ: Non-homologous end joining; PAI-1: Plasminogen activator inhibitor type 1; PARP-1: Poly(ADP-Ribose) polymerase 1; PARPi: PARP inhibitor; PCR: Polymerase chain reaction; PR: Progesterone receptor; SSB: Single-strand breaks; TN: Triple negative breast cancers; uPA: urokinase-type plasminogen activator.

## Competing interests

The authors declare that they have no competing interests.

## Authors’ contributions

WJ participated in the conception and design of the study, provided study patients and material, collected, assembled and interpreted the data and drafted the manuscript. ST participated in the conception and design of the study, participated in the design of the study and performed the statistical analysis. RS collected and assembled the data and carried out the assays. CV collected and assembled the data and participated in the assays. ACL collected and assembled the data and carried out the assays. ELC collected and assembled the data and carried out the assays. FB provided study patients and material, collected and assembled the data. JPB participated in the conception and design of the study and helped to draft the manuscript. GR participated in the conception and design of the study, provided study patients and material and helped to draft the manuscript. PJL participated in the conception and design of the study, provided study material, participated in the assays, collected, assembled and interpreted the data and helped to draft the manuscript. All authors read and approved the final manuscript.

## Pre-publication history

The pre-publication history for this paper can be accessed here:

http://www.biomedcentral.com/1471-2407/13/523/prepub

## Supplementary Material

Additional file 1: Table S1 Patients and Tumours Characteristics of the 18 breast cancers with *BRCA1* promoter methylation. Click here for file

Additional file 2: Figure S1 Correlation between PARP-1 activity and 53BP1 levels. Click here for file

Additional file 3: Table S2 Patients and Tumours Characteristics of the 48 triple negative breast cancers. Click here for file
