# Supplementary material for: BRCA1 promoter hypermethylation, 53BP1 protein expression and PARP-1 activity as biomarkers of DNA repair deficit in breast cancer
Source: BMC Cancer. 2013 Nov 5;13:523. doi: 10.1186/1471-2407-13-523 (PMC4228368; doi:10.1186/1471-2407-13-523)
Supplement: Additional file 1: Table S1 — Patients and Tumours Characteristics of the 18 breast cancers with BRCA1 promoter methylation. [file 1471-2407-13-523-S1.docx]

**Supplementary Table 1**: Patients and Tumours Characteristics of the 18 breast cancers with *BRCA1* promoter methylation

| **Pt Nb** | **Age** | **Histology** | **T status** | **N status** | **Grade** | **Tub. Scor** | **Nucl. Score** | **Mit. Score** | **LVI** | **uPA** | **PAI-1** | **PARP-1** | **53BP1** | **MGP** |
| --- | --- | --- | --- | --- | --- | --- | --- | --- | --- | --- | --- | --- | --- | --- |
| 1 | 38 | Ductal | 1 | 0 | 3 | 3 | 3 | 3 | ND | 5.2 | 13.2 | 6.1 | 3.7 | TN |
| 2 | 43 | Ductal | 1 | 0 | 3 | 3 | 3 | 3 | No | 5.8 | 55.1 | 13.9 | 31.3 | TN |
| 3 | 60 | Other | 2 | 0 | 3 | 3 | 3 | 3 | Yes | 3.1 | 49.9 | 1.8 | 2.0 | TN |
| 4 | 55 | Ductal | 2 | 0 | 3 | 3 | 2 | 3 | ND | 5.7 | 43.9 | 3.5 | 2.0 | TN |
| 5 | 51 | Ductal | 2 | 0 | 3 | 3 | 3 | 3 | No | 6.8 | 16.8 | 9.8 | 3.7 | TN |
| 6 | 48 | Ductal | 1 | N+ | 3 | 3 | 2 | 3 | Yes | 11 | 54.9 | 14 | 2.0 | TN |
| 7 | 54 | Ductal | 1 | 0 | 3 | 3 | 3 | 3 | No | 4.7 | 20.3 | 31 | 2.0 | TN |
| 8 | 61 | Other | 2 | 0 | 3 | 3 | 3 | 2 | No | 5.4 | 100 | 4 | 2.0 | TN |
| 9 | 57 | Ductal | 1 | N+ | 2 | 3 | 2 | 2 | Yes | 3.1 | 21.6 | 2 | 25.9 | HR+/HER-2- |
| 10 | 47 | Ductal | 1 | 0 | 2 | 2 | 3 | 1 | Yes | 5 | 23.8 | 1 | 2.9 | HR+/HER-2- |
| 11 | 31 | Ductal | 2 | N+ | 3 | 3 | 3 | 3 | Yes | 6.7 | 26.4 | 1 | 53.2 | TN |
| 12 | 52 | Ductal | 2 | N+ | 3 | 3 | 3 | 2 | No | 7.4 | 74 | 21 | 10.2 | HER-2+ |
| 13 | 51 | Ductal | 1 | 0 | 3 | 3 | 3 | 2 | No | 8.6 | 55.6 | 5 | 8.9 | TN |
| 14 | 65 | Other | 1 | 0 | 3 | 3 | 3 | 2 | No | 3.2 | 32.8 | 29 | 9.2 | TN |
| 15 | 30 | Ductal | 2 | 0 | 3 | 3 | 3 | 3 | Yes | 2.3 | 17.8 | 29 | 7.8 | TN |
| 16 | 49 | Ductal | 2 | 0 | 3 | 3 | 3 | 3 | No | 2.9 | 35.1 | 15 | 5.7 | TN |
| 17 | 63 | Ductal | 2 | 0 | 3 | 3 | 3 | 3 | Yes | 6.5 | 22.2 | 3 | 2.5 | HR+/HER-2- |
| 18 | 39 | Ductal | 1 | N+ | 3 | 3 | 3 | 3 | No | 14.8 | 28.4 | 3 | 25.5 | TN |

**Abbreviations:** Pt Nb, Patient’s Number; Grade, Elston and Ellis modified Scarff Bloom and Richardson Score (EE-SBR); Tub. score, Tubule formation score from the EE-SBR score; Nucl. score, Nuclear pleomorphism score from the EE-SBR score; Mit. score, Mitotic count score from the EE-SBR score; LVI, LymphoVascular Invasion; BRCA meth, *BRCA1* promoter hypermethylation; MGP, Molecular Grouping Profile; TN, Triple Negative.
