# Supplementary figures and images for: BRCA1 promoter hypermethylation, 53BP1 protein expression and PARP-1 activity as biomarkers of DNA repair deficit in breast cancer
Source: BMC Cancer. 2013 Nov 5;13:523. doi: 10.1186/1471-2407-13-523 (PMC4228368; doi:10.1186/1471-2407-13-523)

## Slide 1
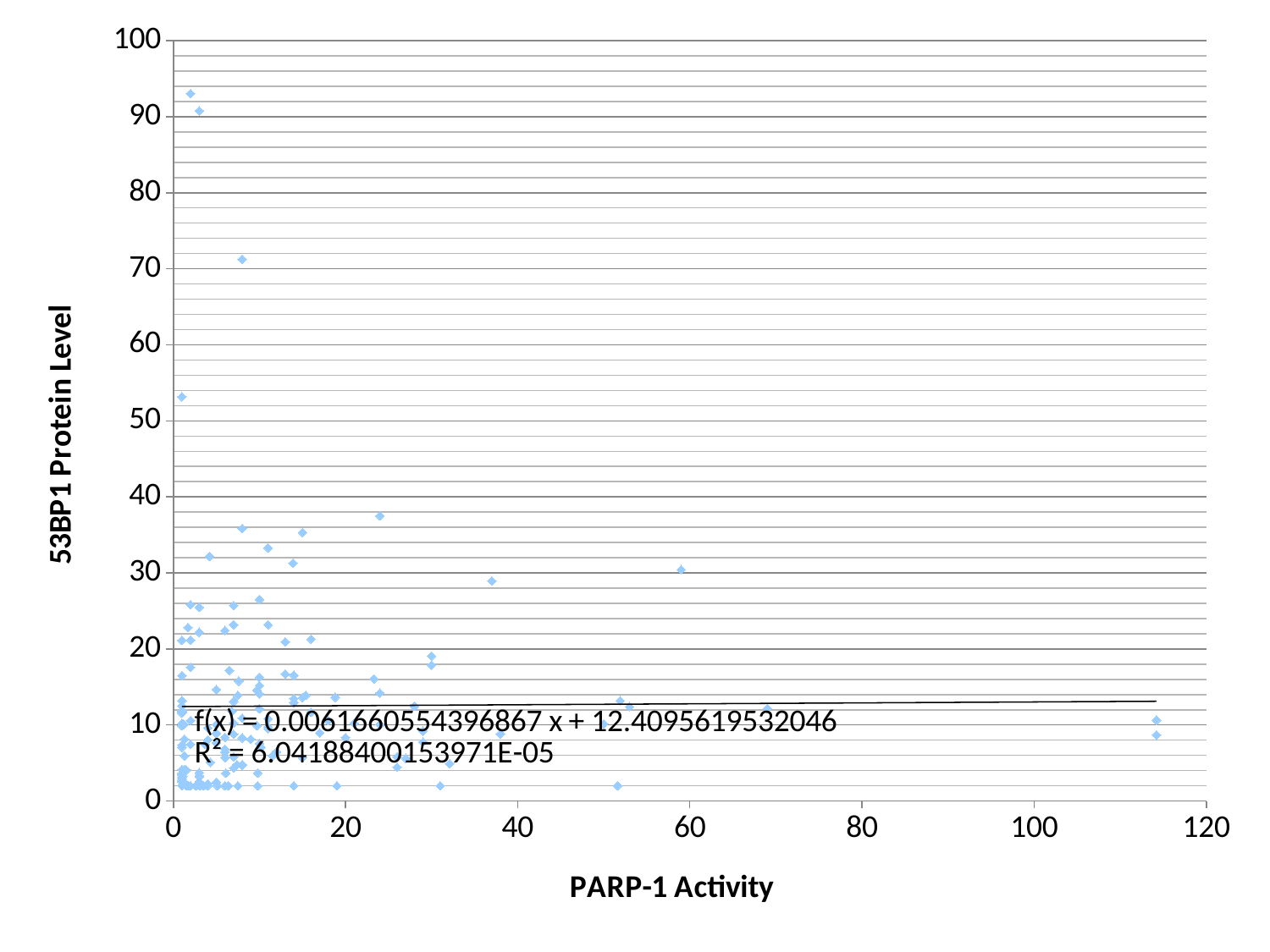

### Chart
| Category | tp53bp1 |
|---|---|#

Supplement: Additional file 2: Figure S1 — Correlation between PARP-1 activity and 53BP1 levels. [file 1471-2407-13-523-S2.pptx]
