# Supplementary material for: BRCA1 promoter hypermethylation, 53BP1 protein expression and PARP-1 activity as biomarkers of DNA repair deficit in breast cancer
Source: BMC Cancer. 2013 Nov 5;13:523. doi: 10.1186/1471-2407-13-523 (PMC4228368; doi:10.1186/1471-2407-13-523)
Supplement: Additional file 3: Table S2 — Patients and Tumours Characteristics of the 48 triple negative breast cancers. [file 1471-2407-13-523-S3.docx]

**Supplementary Table 2**: Patients and Tumours Characteristics of the 48 triple negative breast cancers

| **Pt Nb** | **Age** | **Histology** | **T status** | **N status** | **Grade** | **Tub. Scor** | **Nucl. Score** | **Mit. Score** | **LVI** | **uPA** | **PAI-1** | **BRCA meth** | **PARP-1** | **53BP1** |
| --- | --- | --- | --- | --- | --- | --- | --- | --- | --- | --- | --- | --- | --- | --- |
| 1 | 47 | Other | 2 | 0 | 3 | 3 | 3 | 3 | ND | 1.9 | 200 | N | 1.5 | 2.0 |
| 2 | 61 | Ductal | 2 | 0 | 3 | 3 | 3 | 3 | Y | 2.3 | 6.3 | N | 38 | 8.8 |
| 3 | 38 | Ductal | 1 | 0 | 3 | 3 | 3 | 3 | ND | 5.2 | 13.2 | Y | 6.1 | 3.7 |
| 4 | 47 | Other | 1 | 0 | 2 | 3 | 3 | 1 | N | 6.9 | 9.2 | N | 2.7 | 2.0 |
| 5 | 49 | Ductal | 2 | 0 | 3 | 3 | 3 | 3 | N | 10.8 | 34.3 | N | 1.7 | 22.8 |
| 6 | 43 | Ductal | 1 | 0 | 3 | 3 | 3 | 3 | N | 5.8 | 55.1 | Y | 13.9 | 31.3 |
| 7 | 60 | Other | 2 | 0 | 3 | 3 | 3 | 3 | Y | 3.1 | 49.9 | Y | 1.8 | 2.0 |
| 8 | 62 | Ductal | 1 | 0 | 3 | 3 | 3 | 3 | N | 11.8 | 36.8 | N | 1 | 11.7 |
| 9 | 60 | Ductal | 1 | 0 | 3 | 3 | 3 | 3 | Y | 1.9 | 330 | N | 1 | 7.1 |
| 10 | 62 | Ductal | 1 | 0 | 3 | 3 | 3 | 3 | N | 4.9 | 27.1 | N | 9.7 | 9.9 |
| 11 | 37 | Ductal | 2 | 0 | 2 | 3 | 2 | 1 | Y | 1.7 | 11.9 | N | 1.5 | 4.1 |
| 12 | 63 | Ductal | 1 | 0 | 3 | 3 | 2 | 3 | N | 3 | 12.1 | N | 114.2 | 8.7 |
| 13 | 50 | Ductal | 2 | 0 | 3 | 3 | 2 | 3 | N | 5.7 | 80 | N | 9.8 | 2.0 |
| 14 | 55 | Ductal | 2 | 0 | 3 | 3 | 2 | 3 | ND | 5.7 | 43.9 | Y | 3.5 | 2.0 |
| 15 | 51 | Ductal | 2 | 0 | 3 | 3 | 3 | 3 | N | 6.8 | 16.8 | Y | 9.8 | 3.7 |
| 16 | 62 | Ductal | 1 | 0 | 3 | 3 | 3 | 3 | Y | 9 | 100 | N | 4.3 | 5.1 |
| 17 | 75 | Ductal | 2 | 0 | 2 | 2 | 3 | 2 | Y | 4.4 | 26.3 | N | 32.1 | 4.9 |
| 18 | 63 | Other | 1 | 0 | 2 | 3 | 2 | 1 | N | 1.2 | 47.3 | N | 6.5 | 17.2 |
| 19 | 65 | Other | 2 | 0 | 3 | 3 | 3 | 3 | N | 3.3 | 13.8 | N | 11.5 | 5.9 |
| 20 | 64 | Other | 2 | + | 3 | 3 | 3 | 2 | N | 2.1 | 12 | N | 10.2 | 7.1 |
| 21 | 55 | Other | 1 | 0 | 3 | 3 | 3 | 3 | Y | 2.2 | 5.9 | N | 6.4 | 2.0 |
| 22 | 57 | Lobular | 1 | 0 | 2 | 2 | 2 | 2 | N | .5 | 2.6 | N | 19 | 2.0 |
| 23 | 48 | Ductal | 1 | + | 3 | 3 | 2 | 3 | Y | 11 | 54.9 | Y | 14 | 2.0 |
| 24 | 54 | Ductal | 1 | 0 | 3 | 3 | 3 | 3 | N | 4.7 | 20.3 | Y | 31 | 2.0 |
| 25 | 60 | Ductal | 1 | + | 3 | 3 | 3 | 2 | Y | 1.9 | 21.1 | N | 6 | 22.4 |
| 26 | 61 | Other | 2 | 0 | 3 | 3 | 3 | 2 | N | 5.4 | 100 | Y | 4 | 2.0 |
| 27 | 57 | Other | 3/4 | + | 3 | 3 | 3 | 2 | Y | 7 | 61 | N | 14 | 13.5 |
| 28 | 51 | Ductal | 1 | 0 | 3 | 2 | 3 | 3 | N | 6.3 | 42.9 | N | 2 | 7.4 |
| 29 | 55 | Ductal | 1 | + | 3 | 3 | 3 | 2 | Y | 3.7 | 32.4 | N | 27 | 5.6 |
| 30 | 36 | Ductal | 1 | 0 | 3 | 3 | 3 | 3 | N | 11.7 | 87 | N | 2 | 2.0 |
| 31 | 46 | Ductal | 1 | + | 3 | 3 | 3 | 3 | Y | 2.8 | 20.5 | N | 6 | 2.0 |
| 32 | 41 | Other | 1 | + | 1 | 1 | 2 | 1 | N | 7.8 | 9.6 | N | 1 | 16.5 |
| 33 | 41 | Other | 2 | 0 | 3 | 3 | 3 | 3 | N | 2.4 | 37.8 | N | 37 | 30 |
| 34 | 64 | Ductal | 2 | 0 | 3 | 3 | 2 | 3 | N | 6.2 | 64 | N | 12 | 6.5 |
| 35 | 60 | Ductal | 3/4 | + | 3 | 2 | 3 | 3 | Y | 4.3 | 32.3 | N | 10 | 12.1 |
| 36 | 31 | Ductal | 2 | + | 3 | 3 | 3 | 3 | Y | 6.7 | 26.4 | Y | 1 | 53.2 |
| 37 | 50 | Ductal | 2 | 0 | 3 | 3 | 3 | 3 | N | 6.4 | 91.6 | N | 24 | 14.2 |
| 38 | 51 | Ductal | 1 | 0 | 3 | 3 | 3 | 2 | N | 8.6 | 55.6 | Y | 5 | 8.9 |
| 39 | 43 | Ductal | 2 | + | 3 | 2 | 3 | 3 | Y | 2.8 | 14.6 | N | 3 | 90.8 |
| 40 | 65 | Other | 1 | 0 | 3 | 3 | 3 | 2 | N | 3.2 | 32.8 | Y | 29 | 9.2 |
| 41 | 30 | Ductal | 2 | 0 | 3 | 3 | 3 | 3 | Y | 2.3 | 17.8 | Y | 29 | 7.8 |
| 42 | 66 | Other | 1 | 0 | 2 | 3 | 3 | 1 | N | 4 | 18 | N | 8 | 71.3 |
| 43 | 49 | Ductal | 2 | 0 | 3 | 3 | 3 | 3 | N | 2.9 | 35.1 | Y | 15 | 5.7 |
| 44 | 29 | Ductal | 1 | + | 3 | 3 | 3 | 3 | N | 4.2 | 21.2 | N | 30 | 17.9 |
| 45 | 63 | Ductal | 1 | + | 3 | 3 | 3 | 3 | Y | 8.6 | 28.6 | N | 15 | 13.6 |
| 46 | 53 | Ductal | 2 | + | 3 | 3 | 3 | 3 | Y | 7 | 66 | N | 1 | 2.0 |
| 47 | 39 | Ductal | 1 | + | 3 | 3 | 3 | 3 | N | 14.8 | 28.4 | Y | 3 | 25.5 |
| 48 | 61 | Ductal | 2 | + | 3 | 3 | 3 | 2 | N | 3.9 | 125 | N | 7 | ND |

**Abbreviations:** Pt Nb, Patient’s Number; Grade, Elston and Ellis modified Scarff Bloom and Richardson Score; Tub. score, Tubule formation score; Nucl. score, Nuclear pleomorphism score; Mit. score, Mitotic count score; LVI, LymphoVascular Invasion; BRCA meth, *BRCA1* promoter hypermethylation; Other, other histological subtypes (3 undifferentiated carcinomas, 1 invasive papillary carcinoma, 2 mixed ductal-lobular carcinomas, 1 apocrine carcinoma, 2 medullary carcinomas, 2 metaplastic carcinomas, 2 sarcomatoid carcinomas).
